# Movement Patterns for a Critically Endangered Species, the Leatherback Turtle (*Dermochelys coriacea*), Linked to Foraging Success and Population Status

**DOI:** 10.1371/journal.pone.0036401

**Published:** 2012-05-16

**Authors:** Helen Bailey, Sabrina Fossette, Steven J. Bograd, George L. Shillinger, Alan M. Swithenbank, Jean-Yves Georges, Philippe Gaspar, K. H. Patrik Strömberg, Frank V. Paladino, James R. Spotila, Barbara A. Block, Graeme C. Hays

**Affiliations:** 1 Chesapeake Biological Laboratory, University of Maryland Center for Environmental Science, Solomons, Maryland, United States of America; 2 National Oceanic and Atmospheric Administration/National Marine Fisheries Service/SWFSC/Environmental Research Division, Pacific Grove, California, United States of America; 3 Department of Biosciences, College of Science, Swansea University, Singleton Park, Swansea, United Kingdom; 4 Center for Ocean Solutions, Monterey, California, United States of America; 5 Hopkins Marine Station, Stanford University, Pacific Grove, California, United States of America; 6 Université de Strasbourg, IPHC, Strasbourg, France; 7 CNRS, UMR7178, 67037 Strasbourg, France; 8 Department of Marine Ecosystems, Collecte Localisation Satellites, Satellite Oceanography Division, Ramonville St Agne, France; 9 Swedish Meteorological and Hydrological Institute, Folkborgsvägen 1, Norrköping, Sweden; 10 Department of Biology, Indiana-Purdue University, Fort Wayne, Indiana, United States of America; 11 Department of Biology, Drexel University, Philadelphia, Pennsylvania, United States of America; University of Alberta, Canada

## Abstract

Foraging success for pelagic vertebrates may be revealed by horizontal and vertical movement patterns. We show markedly different patterns for leatherback turtles in the North Atlantic versus Eastern Pacific, which feed on gelatinous zooplankton that are only occasionally found in high densities. In the Atlantic, travel speed was characterized by two modes, indicative of high foraging success at low speeds (<15 km d^−1^) and transit at high speeds (20–45 km d^−1^). Only a single mode was evident in the Pacific, which occurred at speeds of 21 km d^−1^ indicative of transit. The mean dive depth was more variable in relation to latitude but closer to the mean annual depth of the thermocline and nutricline for North Atlantic than Eastern Pacific turtles. The most parsimonious explanation for these findings is that Eastern Pacific turtles rarely achieve high foraging success. This is the first support for foraging behaviour differences between populations of this critically endangered species and suggests that longer periods searching for prey may be hindering population recovery in the Pacific while aiding population maintenance in the Atlantic.

## Introduction

Foraging success is intimately linked to reproductive success and hence population viability [1], [2]. The processes that drive foraging success may therefore strongly shape the conservation status of populations. An ecological and conservation enigma has existed for critically endangered leatherback turtles (*Dermochelys coriacea*), which despite facing high fisheries bycatch across the world’s oceans [3], [4], show markedly different population trajectories in the Atlantic and the Pacific [5], [6]. Leatherback turtles in the Pacific Ocean have been rapidly decreasing over the past two decades, whereas those in the North Atlantic are stable or increasing.

It is well known that foraging success for pelagic vertebrates may be revealed by horizontal movement patterns [7]. Predators tend to focus their foraging attention on areas where they have recently encountered prey by reducing their speed and/or increasing their turning angle. This behaviour is known as area-restricted search (ARS) and results in populations of predators moving toward regions of high prey density [8]. The elucidation of movement patterns through electronic tagging has enabled spatio-temporal foraging patterns to be determined for many pelagic species [9], [10]. A significant negative relationship between travel speed and the number of feeding events has also been found in a large marine predator [10]. In an analysis of foraging success measures, travel rate consistently provided the best estimate of daily foraging success [11].

In this study, we analysed the movement patterns, and more specifically travel rates, of North Atlantic (NA) and Eastern Pacific (EP) leatherback turtles derived from Argos satellite tracks to determine if there were differences in their foraging behaviour. Since high density aggregations of gelatinous zooplankton, upon which leatherbacks feed, are patchily distributed [12], leatherbacks would only occasionally be expected to find themselves in high density prey fields. So *a priori* one would expect that leatherbacks would spend most of their time transiting in search of high density prey patches [13]. Rapid movements away from the breeding areas and during their seasonal migratory cycle suggest replenishing their energy reserves quickly is important [14], [15]. We therefore predict that for this species the modal speed will represent movement associated with prey search. When high density prey patches are located, leatherbacks remain in the area to feed and their travel speed decreases. Accordingly, we hypothesize that the declining EP leatherbacks may have lower foraging success than those in the NA, and that the travel rates will correspondingly be different between the two populations. As changes in vertical movements may also be indicative of foraging [15], we complemented our analysis of horizontal movement patterns with a comparison of diving behaviour by NA and EP leatherback turtles. We used this comparison of movement patterns to identify differences in foraging behaviour between leatherbacks in the Atlantic and Pacific and how this relates to the population abundance trends.

## Methods

### Ethics Statement

The study adhered to the legal requirements of the countries in which the work was carried out, and to all institutional guidelines. Fieldwork in Grenada was carried out with approval from the Ministry of Agriculture, Forestry, Lands and Fisheries of Grenada with permission granted under the Fisheries Act #15 of 1986 (section 24) and the Fisheries Regulations SRO # 9 of 1987 (section 21). In Ireland all tagging was carried out under the strict guidance and approval of the National Parks and Wildlife Service of the Department of Environment, Heritage and Local Government, Ireland. Fieldwork in French Guiana and Suriname was carried out under CNRS-IPHC institutional license (B67 482 18) and individual licences to JYG (67–220 and 04–199) and SF (67–256) delivered by the National Committee of Nature Protection (French Ministry of Ecology and Sustainable Management), Paris, France; the Departmental Direction of the Veterinary Services, Strasbourg, France; and the Police Prefectures of Bas-Rhin and French Guiana. The Eastern Pacific leatherback tracking research was approved by the Stanford University Research Compliance Office Administrative Panel on Laboratory Animal Care under Protocol #13848: ‘Satellite tracking of Eastern Pacific leatherback sea turtles’. Permits were obtained via Resoluciones 273-2003-OFAU, ACT-OR-056, y ACT-OR-032-06 from the Costa Rican Ministerio de Ambiente y Energia y Telecomunicaciones (MINAET) for Estudios de la conducta, movimientos, y uso de hábitat de las Tortugas Baulas (*Dermochelys coriacea*) en el Parque Nacional Marino Las Baulas, Área de Conservación Tempisque.

### Data Collection

Our study involved a synthesis of some previously published tracks [9], [16]. ARGOS-derived surface locations were obtained from 46 EP leatherback turtles [16]. These turtles were tagged during nesting at Playa Grande, Costa Rica in 2004 to 2007. In 2004, Wildlife Computer Smart Position Only tags (SPOT) were attached to 10 of these turtles. The remaining turtles were instrumented with Sea Mammal Research Unit (SMRU) Satellite Relay Data Loggers (SRDL). The NA leatherback turtles were tagged during 2002 to 2006 at Levera beach in Grenada (n = 9), Samsambo beach in Suriname (n = 1), Awala-Yalimapo beach in French Guiana (n = 5), at sea off Nova Scotia (n = 4) and off the Dingle Peninsula in County Kerry, Ireland (n = 2) [9]. These turtles were all fitted with SMRU SRDL tags. The satellite transmitters were attached to all of the turtles in the study with a harness system that had corrodible links for release [17], [18], except for three NA turtles that had the SRDLs directly attached to the carapace [19]. Since turtles tagged with harnesses have been found to travel slower than those directly attached [19], the three tracks with direct attachment were excluded from further analysis.

### Track Analysis

Details of the processing of the NA leatherback tracks are given in Fossette et al. [9]. Briefly, locations of all location quality classes were analysed, but those with an apparent speed >10 km h^−1^ were discarded as they were considered biologically unlikely [15]. Tracks were then smoothed and re-sampled to provide positions at regular intervals [9].

The switching state-space model (SSSM) developed by Jonsen et al. [20] was applied to all of the raw surface positions of the EP leatherback turtle tracks. The application of the SSSM provided the most probable track positions, taking into account Argos location error, at regular 6 h intervals [16], [21].

In this study, we only used the post-nesting portions of all of the tracks, after nesting had been completed. Travel speeds were calculated from the regularised tracks via first differencing consecutive points. Track sections were removed where there had been more than 3 days missing satellite data as this could result in an underestimate of travel rate [9]. Frequency distributions were generated for the absolute travel speeds. A Hartigan’s dip test was performed to determine whether the distributions were unimodal [22].

We tested whether the processing of the satellite data affected the travel speeds or had any impact on the frequency distributions. We applied the SSSM method to one of the NA tracks and compared the mean speeds between the two processing methods. The SSSM may have filtered out more of the high speed variations resulting in a unimodal distribution for the EP population. We therefore also applied a simple filtering process to check whether the distribution of travel speeds for the EP turtle tracks was the same regardless of the processing method. This simple filter removed segments where speeds were >10 km h^−1^, similar to the method by Fossette et al. [9], and Z class positions. A Hartigan’s dip test for unimodality was then applied.

The two modes identified in the NA absolute travel speed distribution were at 12.5 km d^−1^ and 37.5 km d^−1^ (Figure S1). Around each of these modes 29% and 42% of the time was spent travelling between 0–15 km d^−1^ and 20–45 km d^−1^ respectively. Given that leatherbacks are presumed to spend most of their time transiting in search of prey and relatively little time achieving high foraging success [13], we defined the modal transiting speed as 37.5 km d^−1^ for the NA leatherbacks based on the modal speed that encompassed most of the data. A single peak modal speed was identified for the EP turtles at 21 km d^−1^. Frequency distributions for the relative travel speeds were generated accordingly. The SSSM unfortunately classified large portions of the 6-hourly positions as a single behavioural mode [21], which made it difficult to identify specific foraging areas. The comparison of travel speeds therefore appeared more robust and highlighted more clearly the movement differences between the two populations.

We also plotted frequency distributions of the travel speeds for subsets of the data. The NA turtles were tagged both on the nesting and foraging grounds. We therefore selected the speeds from only those NA turtles tagged with harnesses on the nesting grounds so that they would be more directly comparable with those of the EP turtles, which were only tagged in this way. The NA turtles also have three main migration strategies that were plotted separately: the round-trip, the northern and the equatorial [9]. Finally, within the EP tracking data set there was a single coastal forager. The speeds for this turtle were plotted to compare with the migration strategies by the NA turtles.

### Track Current Correction

The impact of the ocean currents on each turtle’s trajectory was removed to reveal the animal’s swimming velocity [9], [23]. The ocean currents were calculated using surface current estimates measured by satellite that are the sum of the geostrophic and Ekman components. These surface currents were deducted from the turtle movements at each location. A β-plane solution was applied within the equatorial band (4°N – 4°S) [16].

### Comparison with Chlorophyll-a Concentration

Very little is known about the distribution and abundance of leatherback turtle prey, gelatinous zooplankton, and data are particularly sparse in the South Pacific Ocean. We therefore used estimates of near-surface chlorophyll-*a* concentration (CHL; an indicator of phytoplankton standing stock) as a proxy for leatherback prey abundance. A long-term mean was calculated from Moderate Resolution Imaging Spectroradiometer (MODIS) satellite ocean-color observations at 9 km resolution for the period July 2002 to July 2010. CHL values for the time and location of each turtle position were obtained from 8-day composites provided by the MODIS (0.05° × 0.05° resolution) sensor on the NASA AQUA/TERRA Spacecraft. These were extracted using the OceanWatch Themetic Real-time Environmental Distributed Data System (THREDDS, available at http://oceanwatch.pfel.noaa.gov/thredds). Mean CHL values were calculated for all of the EP turtle positions and for the NA turtle positions where speeds were ≤40% of the modal transiting speed (15 km d^−1^).

### Vertical Behaviour

Changes in vertical movements may also be indicative of foraging [15]. We therefore analysed the vertical behaviour of the turtles derived from the dive data recorded by the tags. These dive data are summarised into 6 h bins [9], [24]. We examined the mean dive depths in relation to latitude for the two populations to provide further insight into where leatherbacks are foraging. The vertical distribution of prey was not available so we calculated the depth of the mean annual thermocline and nutricline. These are indicators of temperature and nutrient changes that influence biological productivity and hence leatherback gelatinous zooplankton prey. The thermocline depth was defined as the depth of the maximum temperature gradient. The nutricline is the increase in concentration of nutrients with depth and was expressed as the concentration of nitrate. The 2 µmol nitrate isocline depth was used as a proxy for the nutricline [25]. These were calculated from the objectively analysed annual climatology of temperature and nitrate values on a 1 degree grid at standard depth levels from the World Ocean Atlas 2009 [26], [27].

## Results

### Horizontal Movements

**Figure 1:**
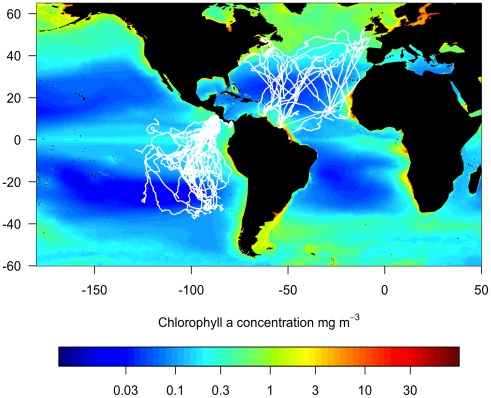
Leatherback turtle satellite tracks. Map of North Atlantic (NA) and Eastern Pacific (EP) leatherback turtle tracks overlaid on a long-term mean (2002–2010) of near-surface chlorophyll-*a* concentration.

The EP leatherback turtles tended to move within a migratory corridor to foraging grounds in the southeast Pacific [16], [28], whereas the NA turtles dispersed throughout the North Atlantic Ocean [29], [30], with foraging behaviour occurring primarily at high latitudes and in the sub-Equatorial region [9] (Figure 1).

There was a 0.5 km d^−1^ difference in the mean speed for the NA turtle track (duration = 323 days) with the Fossette filtering method (Mean = 26.5, SD = 17.1 km d^−1^) and SSSM (Mean = 27.0, SD = 19.3 km d^−1^). The travel speed histogram from the Pacific SSSM-derived positions had the same unimodal distribution (Hartigan’s dip test, P = 0.999) as that derived from positions where a simple location class and speed filter had been applied to the satellite locations (Hartigan’s dip test, P = 0.999) (Figure S1). This indicates that any differences between the populations in the travel speed distributions were not caused by differences in the processing of the satellite data.

**Figure 2:**
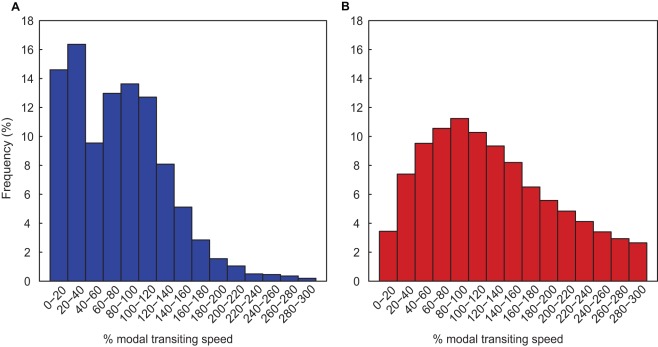
Frequency distribution of percent modal transiting speed for leatherback turtles. Speeds as a percentage of the modal transiting speed in the a) North Atlantic (NA) (modal transiting speed = 37.5 km d^−1^), and b) Eastern Pacific (EP) (modal transiting speed = 21 km d^−1^), showing the bimodal and unimodal distributions respectively.

**Figure 3:**
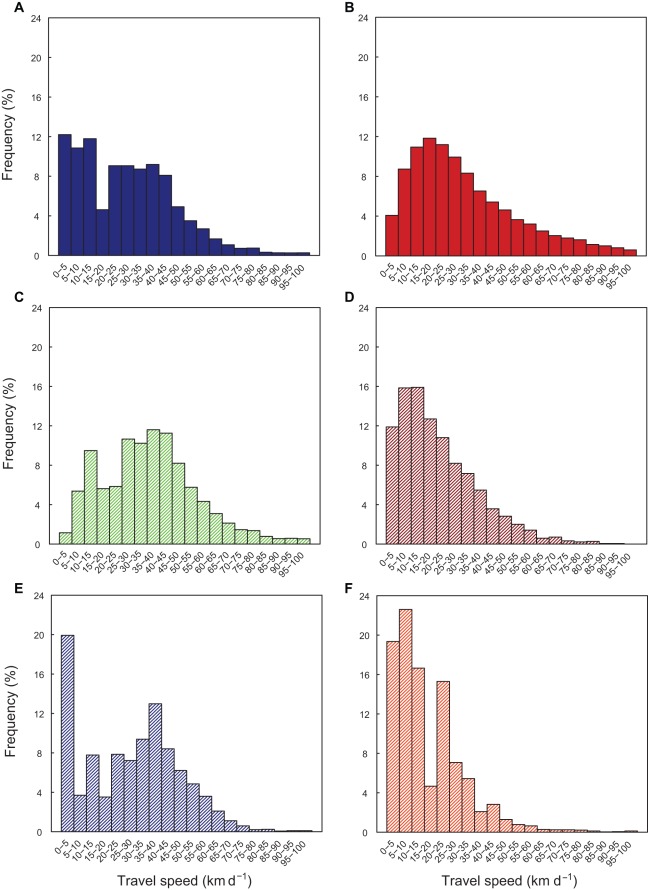
Frequency distributions of leatherback travel speeds. Speeds for turtles harness tagged on the nesting beach in a) the North Atlantic (NA) (n = 13), and b) Eastern Pacific (EP) (n = 46). The speeds for the three main migratory strategies of North Atlantic leatherbacks c) round-trip (n = 11), e) northern (n = 4) and f) equatorial (n = 3), compared with d) the single coastal forager of the EP leatherbacks.

Although mean turtle travel speeds were similar in the Atlantic (Mean = 30.5, SD = 21.3 km d^−1^) and the Pacific (Mean = 34.5, SD = 27.6 km d^−1^), there was a significant difference in the frequency distribution of both absolute and relative travel speeds between the two populations (Figures 2, 3 and S1). The NA travel speeds had a bimodal distribution (Hartigan’s dip test, P<0.001), whereas only a single mode was evident for the EP turtles (Hartigan’s dip test, P = 0.999). Given that leatherbacks are presumed to spend most of their time transiting in search of prey and relatively little time achieving high foraging success [13], we defined the modal transiting speed as 37.5 km d^−1^ and 21 km d^−1^ for the NA and EP leatherbacks respectively. The second mode (12.5 km d^−1^) identified in the NA travel speed distribution (Figure 2), at slower speeds and indicative of high foraging success, corresponded to travel speeds ≤40% of the modal transiting speed. There was no corresponding slower speed mode in the EP travel speed distribution and there was a lower frequency of slow speeds (<15 km d^−1^) than in the NA distribution (Figures 2, 3A and B). These distribution patterns also occurred when we used satellite derived current information to remove the impact of the ocean currents on each turtle’s trajectory and hence reveal the animals’ swimming velocity (Figure S2).

Analysis of speeds for only those NA turtles tagged on the nesting ground confirmed the strongly bimodal distribution and the difference between the two populations during the early post-nesting phase (Figure 3A and B). The three migratory strategies of the NA turtles also all had bimodal travel speed distributions (Figure 3C, E and F). In contrast, the coastal forager EP turtle had a single peak like that for the other EP turtles (Figure 3D), but it occurred at slower travel speeds (<15 km d^−1^). This peak was similar to the travel speed of the first peak for the NA turtles.

Slower travel speeds tended to occur at high latitudes for both populations. The locations of slow travel in the North Atlantic (≤40% of the modal transiting speed) had a mean near-surface chlorophyll-*a* concentration (CHL) of 0.67 mg m^–3^ (SD = 1.83, Range = 0.04–19.86 mg m^–3^). If we consider the EP turtles as constantly searching for prey as they had a unimodal travel speed distribution, the mean CHL at their locations was 0.18 mg m^–3^ (SD = 0.34, Range = 0.01–6.30 mg m^–3^). This is less than a third of that for the foraging NA turtles. This was still true even when we considered only the locations at ≤40% (8.4 km d^−1^) of the modal transiting speed for EP turtles (mean CHL = 0.22, SD = 0. 61 mg m^–3^). Leatherback turtles feed on gelatinous zooplankton and CHL is therefore used only as a proxy for prey biomass. Estimates of global zooplankton biomass show similar patterns to CHL [31]. Emerging global estimates of gelatinous zooplankton biomass [32] may lead to better understanding of the prey field for leatherback turtles.

### Vertical Behaviour

**Figure 4:**
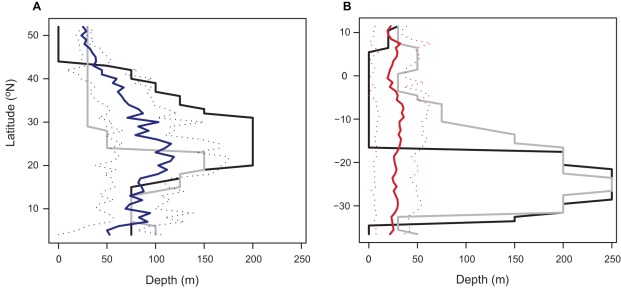
Depth distribution in relation to latitude. Mean (± SD) leatherback turtle dive depth (solid and dotted blue and red lines), mean annual thermocline (gray) and nutricline (black) depth in relation to latitude (1° bins) in the a) North Atlantic (NA), and b) Eastern Pacific (EP).

There were greater depths and variation in the mean dive depth with latitude for the NA turtles than the EP turtles (Figure 4). The NA leatherbacks dove deeper at mid-latitudes, which is where the nutricline and thermocline were also deepest. Their mean dive depth was shallowest at low and high latitudes. At high northerly latitudes, their mean depth was below the mean annual nutricline depth and very close to the mean annual thermocline depth. The EP leatherbacks similarly dove below the nutricline and close to the thermocline depth at high latitudes. This also occurred within the equatorial band. However, the EP turtles had relatively shallow dive depths at all latitudes and did not follow as strong a pattern as the thermocline and nutricline of increasing depth at mid-latitudes.

## Discussion

We identified different movement patterns for leatherback turtles in the Atlantic versus Pacific Ocean. The most parsimonious explanation for this finding is that the lack of a mode at slower travel speeds for EP leatherback turtles is because they rarely achieve high foraging success and spend most of their time transiting in search of prey; while for NA leatherbacks the faster mode in travel speed may be indicative of transiting and the mode at relatively slow speeds is caused by turtles staying within high density prey patches [13]. These travel speed distributions provide the first support for a difference in foraging behaviour between leatherbacks in the Pacific versus Atlantic, and suggest that intrinsic properties of the foraging habitat may be hindering population recovery in the Pacific while aiding population maintenance in the Atlantic. Foraging movements, indicated by lower travel speeds and changes in dive behaviour [9], [13], [24], have previously been associated with upwelling and oceanic fronts that can concentrate prey [29], [33]. The generally lower CHL values in the southeastern Pacific Ocean (Figure 1) indicate that there may be lower abundances of gelatinous zooplankton and/or that they are more widely dispersed. The EP leatherbacks may therefore be spending longer periods of time searching for food. This provides further evidence that resource limitation may be a contributing factor to the longer intervals between nesting events (remigration) and to the downward population trend [34], [35].

Leatherbacks tended to dive to shallower depths at low and high latitudes (Figure 4). Dive durations were similarly shorter at these latitudes and accompanied by changes in diel dive activity [13], [24]. These changes in diving behaviour suggest that foraging predominantly occurs within the equatorial and high latitude regions, which corresponds with the edges of the North Atlantic and South Pacific Gyres [9], [16]. Estimates of zooplankton biomass are generally higher around the edges of these gyres, in upwelling regions and on the continental shelf [31]. Gelatinous zooplankton also frequently accumulate around physical discontinuities, such as thermoclines [12]. High values of zooplankton biomass have been observed where the depth of the thermocline is shallow [36]. This would explain the shallower mean dive depths by leatherbacks when the thermocline and nutricline were also both shallow. The thermocline and nutricline play an important role in controlling vertical nutrient fluxes, which affects primary production [37]. This is turn increases production higher up in the food web, including the abundance of gelatinous zooplankton, and consequently leatherbacks. The thermocline and nutricline are substantially deeper in the centre of the South Pacific Gyre than in the North Atlantic Gyre. If prey are aggregated at this depth it could mean it is no longer energetically profitable for turtles to dive so deep to feed, and may explain the lack of an association between the EP dive depths and the thermocline/nutricline in this region. It may also be that productivity is so low and prey items are so scarce in the centre of the South Pacific Gyre [31], [38] that it is not worth diving any deeper to search for prey. Movement above and below the thermocline may additionally be a function of thermoregulation in leatherbacks [39], [40].

The life history strategy of leatherback turtles is characterized by deferred maturity (24.5 to 29 years [41], 16.1 years [42], 12–14 years [43]) and long-life span. Long-lived species are generally characterized by high adult survival, where breeding adults must trade off current versus future reproductive success. When there are limited energy resources, breeding adults may reduce egg size or number, invest a greater proportion of their energy reserves to egg production, delay egg production until later in the season, or wait to breed until the following season [2]. This final strategy is only profitable for long-lived species that have a high probability of surviving to breed in the future. Another long-lived species, the chinstrap penguin (*Pygoscelis antarctica*), has been found to reduce reproductive success rather than increase foraging effort in response to lower prey abundance [44]. The leatherback turtle may therefore be responding to large fluctuations in prey availability in the southeast Pacific, for example caused by the El Niño Southern Oscillation, by holding its foraging effort constant and allowing its reproductive success (nesting probability and number of eggs per clutch) to vary between years [5], [45]. The high adult mortality caused by bycatch in fisheries [46], may be reducing the profitability of this strategy by lowering the probability of future breeding, and hence reducing the population abundance.

The NA turtles, although also having suffered high mortality from bycatch [4], may be able to recover more easily by accessing feeding areas with high prey densities [47]. Many turtles migrated to highly productive areas in the North Atlantic Ocean, including productive coastal waters where gelatinous zooplankton biomass tends to be highest [32] (Figure 1). This may allow them to return more regularly to nest and lay greater numbers of eggs [5], which would compensate for the loss of breeding adults. In contrast, the majority of EP leatherbacks migrated south into the South Pacific Gyre, which is highly oligotrophic. Only one leatherback turtle in the EP tracking data set foraged within coastal waters [16]. Another EP leatherback has been documented migrating from a nesting beach in Mexico to the coast of South America [48]. High rates of leatherback mortality among gillnet fisheries along the Central and South American coasts may have drastically reduced the number of coastal foragers [5], [48]. Consequently, the remaining pelagic foragers may not represent the most efficient foraging strategy as they have long distances to travel to their foraging grounds, and less dense and predictable prey sources.

Using stable isotope analysis, the nitrogen signature of EP leatherbacks indicated that they foraged within the highly denitrified eastern equatorial Pacific, and was very different from that of the NA leatherbacks implying distinct oceanographic processes on their separate foraging grounds [49]. A single nesting Atlantic leatherback population was also segregated into two distinct isotopic groups [50]. This implied differences in their choice of feeding habitats, with an offshore North Atlantic group and a more coastal West African group. A trophic dichotomy has similarly been identified in adult female loggerhead turtles, where oceanic planktivory occurred in small females and neritic benthivory by large females [51]. Energy budget calculations indicated the small oceanic females required almost 17 times longer to accumulate the necessary energy for reproduction than the large benthivorous females, which accounted for the intrapopulation variation in remigration intervals [51]. The relatively small size, long remigration intervals, and oceanic foraging of the EP leatherback females [5], suggests that this may also apply to this population and that the less energetically profitable oceanic strategy is currently dominant. The only coastal forager in the EP tracking data set was one of the largest individuals and had a higher than average clutch size (PTT 56280, Table 1 in [52]), providing further support for this hypothesis. This turtle also travelled slowly and had a peak at speeds similar to the first mode by the NA turtles (Figure 3) indicating high foraging success in this coastal area.

The foraging pattern of EP leatherbacks identified from satellite telemetry in this study may therefore not be the optimal strategy for leatherback turtles in the eastern Pacific Ocean. The longer remigration interval and decline in numbers may be the consequence of a synergistic interaction between environmental (affecting prey resources) and anthropogenic impacts (affecting adult survival rates) [34]. High prey availability at the foraging grounds of the NA leatherbacks [53] may have enabled them to maintain high reproductive success, which has compensated for adult mortality in pelagic longline and gillnet fisheries [4], [54]. Our analysis of travel speeds indicates that the EP leatherbacks are not compensating for low prey availability by increasing their foraging effort or efficiency. This is also supported by a study that showed delayed remigration in the EP population did not result in enhanced growth or measured indices of reproduction, indicating variability of environmental conditions is driving the length of the remigration interval and thus the overall reproductive output for each female [55]. It is essential that efforts to protect nesting beaches are combined with plans to reduce fisheries bycatch so that adult mortality is lowered, improving the profitability of their evolved life history strategy and allowing populations to recover [43].

## Supporting Information

Figure S1 **Leatherback turtle absolute travel speeds.** Frequency distribution of leatherback turtle speeds of travel in the a) North Atlantic (NA), including all harness tagged turtles (n = 18), and b) Eastern Pacific (EP), using a simple speed and location class filter (n = 46).
(EPS)

Figure S2 **Current-corrected leatherback turtle swimming velocities.** Frequency distribution of current-corrected leatherback turtle swimming velocities for a) North Atlantic (NA) leatherbacks, and b) Eastern Pacific (EP) tracks.
(EPS)
